# Effects of Operating Parameters on Ionic Liquid Membrane to Remove Humidity in a Green Continuous Process

**DOI:** 10.3390/membranes9050065

**Published:** 2019-05-24

**Authors:** Xueru Yan, Alexandre Favard, Stéphane Anguille, Marc Bendahan, Philippe Moulin

**Affiliations:** 1 Aix Marseille Univ., Centrale Marseille, CNRS, M2P2 Aix en Provence, France; xueruy@yahoo.com.sg (X.Y.); stephane.anguille@univ-amu.fr (S.A.); 2 Aix Marseille Univ., Université de Toulon, CNRS, IM2NP Marseille, France; alexandre.favard@ecologicsense.fr (A.F.); marc.bendahan@univ-amu.fr (M.B.)

**Keywords:** ionic liquid-based membrane processes, gas separation, removing humidity, operating parameters, membrane regeneration

## Abstract

Membrane processes are promising methods to separate gases from feed streams without phase changing. A hybrid process, the combination of ionic liquids with a ceramic membrane (ILM), has been developed for humidity removal in a green continuous process. This new concept provides a more efficient and available ionic liquid (IL)-based membrane regeneration process, which just switches the moist feed stream to dry air. Furthermore, the ILM presents high stability and mechanical resistance during long-time operation. In addition, the influences of several operating parameters, including flow rate, temperature, absolute pressure, and feed concentration on process efficiency were investigated. The lower inlet flow rate was found to be favorable for drying humid air. Moreover, when the pressure increased, the mass of absorbed water was increased, while the feed concentration had no significant effects on the membrane separation performance. However, the operating temperature had a great effect on humidity removal. It is necessary to note that the processes at room temperature can limit the energy consumption. The absorbing process of ILM remained efficient after several absorption desorption cycles. Therefore, the new ILM hybrid process that has been developed has great potential for consecutive humidity removal processes.

## 1. Introduction

Recently, membrane-based separation processes have aroused researchers’ interest, because membrane processes are driven by a pressure gradient [1], making them greener and more energy-efficient approaches. Ionic liquids (ILs) are organic salts with unique properties, such as negligible vapor pressure, low flammability, and high thermal and chemical stabilities over a wide temperature range [2]. These properties enable ILs to replace traditional organic solvents. Moreover, the applications of ILs are increasing due to their tailor-made properties to fit different applications [3,4,5,6,7]. Currently, the combination of these features has brought opportunities in applications of IL-based membranes. In addition, these processes are used to remove water vapor [8,9], and are believed to be more environmentally friendly and energy-saving compared to other commercial separation processes. 

A new membrane, a combination of ILs and a ceramic membrane (ILM), has been developed [10,11], with the aim of gas separation from feed streams. This new ILM is a multi-functional separating device, which can achieve removing harmful gas or liquid, the separation of biogases, and humidity removal. Comparing to conventional humidity removal processes, ILMs provide high stability and mechanical resistance during long-time operation. Due to its configuration, the ILM process protects the ionic liquid from other compounds in the effluent, such as suspended matter or high organic matter (>1000 g·mol^−1^). A water-loading ILM can be regenerated directly instead of taking absorbents out of the operating system to dry.

Humidity has negative effects on numerous applications. For example, metal oxide gas sensors are widely used to monitor the concentrations of harmful gases, due to their high sensitivity. However, according to the previous studies, the sensitivity of metal oxide sensors can be decreased 50% in the presence of humidity [11,12,13]. For the gas processing, as the temperature and pressure change during the production of the gas, water can condense from the gas stream, which result in the blockage of gas transmission lines [14]. In this case, it is necessary to separate the moisture from the feed streams.

This work presents the application of the hygroscopic ionic liquid 1-butyl-3-methylimidazolium bromide ([bmim]Br) in membrane-based humidity removal processes. Furthermore, the optimal operating conditions in a continuous green separation process are investigated. It is expected that the stability and separation ability of the ILM for humidity removal will be enhanced, due to the strong absorption of IL synergy with membrane separation. The regeneration process of ILM is simple and efficient, and the ILM showed high stability during long-time operation.

## 2. Materials and Methods 

### 2.1. Ionic Liquid Membrane (ILM)

**Figure 1:**
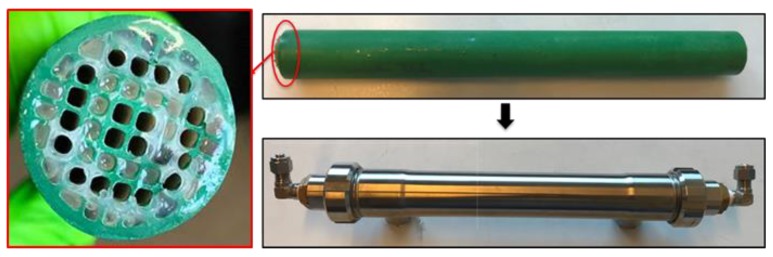
Photos of the combination of an ionic liquid and a ceramic membrane (ILM).

As shown in Figure 1, the ILM is a multichannel tubular ceramic membrane (Alsys Society, Salidares, France), wherein some channels are filled with IL and some channels are empty for gas or liquid access. The channels containing IL are sealed by glue on each extremity, to keep the ionic liquid inside. Before preparing the ILM, the dissolved gases into the selected IL were removed by evaporating. For ILM fabrication, first the channels containing IL were sealed by glue on one extremity, while other channels are sealed by rubber plugs on another extremity. Then IL will be filled into the blocked channels until full. The channels filled with IL will be sealed on another extremity by glue. The glue, which is a mixture of alkylamine (R-NH_2_) and ethylene oxide derivatives (R^1^-CH-O-CH-R^2^), was purchased from Sika (Paris, France), and is not dissolved by the selected IL. The outside of the ceramic membrane is sealed with a hydrophobic coating. The coating used is that (fluoropolymer) which is conventionally used for the ends of the micro-ultra-nanofiltration membranes. The stainless steel carter is used to stabilize ILM and connect the system. The main inventions of our ILM include the following: first, the ILM combines the membrane separation process with the absorption process of ILs; second, this membrane can achieve separation and regeneration processes in the single and same unit; third, the ILM can separate the suspended matters or pollutants from liquid or gas. Full details of the ILM tested are given in Table 1.

### 2.2. Ionic Liquids (ILs)

Among the available ILs, 1-butyl-3-methylimidazolium bromide ([bmim]Br), 1-butyl-3-methylimidazolium hexafluorophosphate ([bmim][PF_6_]), and trihexyl(tetradecyl)phosphonium chloride ([3htdp]Cl) were selected in this study for humidity removal. All ILs used in this article were purchased from Solvionic (Toulouse, France). They have been chosen for their hydrophilic–hydrophobic behaviors and their viscosity properties. The chemical structures of the ILs are shown in Table 2. As far as the selection of IL was concerned, water solubility was an important factor for consideration. The rate of water solubility in an IL is governed by many factors, including the operating pressure and temperature, and chemical structure of IL [15,16,17,18]. According to previous studies, the solubilities of an IL are primarily defined by the anion, followed by the cation alkyl side chain length. With regard to cations, short and mono-branched alkyl chains are recommended for increasing the miscibility of ILs with water. A higher electronic acidity of the cation is preferable for achieving higher water solubility [1,2,15,16]. Other than that, [bmim]Br could promote a greater solubility of water. The ILs were used after removing dissolved gases under a vacuum. The effects of different parameters, including the concentration of the inlet relative humidity, flow rate, pressure, and temperature were investigated in a green continuous process.

The saturated masses of water absorbed by each IL were tested under room temperature and atmospheric pressure in an open flask. The flasks contained different ILs (10 g) that were put in a humid condition (relative humidity (RH) = 90%). In addition, the weights of the flasks were recorded each week until the weights remained constant. The additional weight of each flask is regarded as the maximum water absorbed by ILs. The results were exhibited in Table 2.

### 2.3. Experimental Set-Up

**Figure 2:**
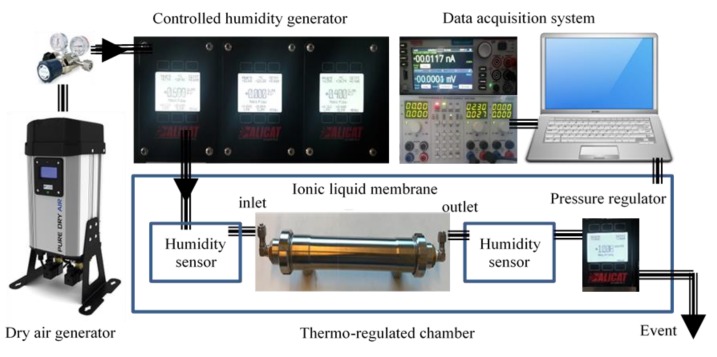
The schematic diagram of the test bench.

The test bench (Figure 2) consists of a zero-air generation system coupled with a humidification system. It allows the generation of humid air in a range between 0 and 90% relative humidity. The air flow is controlled by a mass flow meter between 0.05 and 2.00 L·min^−1^. The operating pressure can be adjusted by a digital pressure regulator. The membrane is inserted into a thermoregulated chamber to control the operating temperature and to optimize the process. 

Humidity is generated starting from pressurized liquid water, which is vaporized through a microporous membrane. The water vapor is injected into the dry gas mixture by means of a proportional valve. This valve, controlled by a humidity sensor placed at the humidifier outlet, makes it possible to keep the hygrometry of the mixture constant. The vapor pressure is kept sufficient by heating and regulating the temperature of the vaporization cell. A second humidity sensor is placed at the output of the ILM. A computer is used to control the process and to acquire moisture measurements continuously over time.

### 2.4. Analysis of the Results

In our system, all the inlet parameters were constant, including flow rate, inlet humidity, pressure, and temperature. The levels of humidity after the ILM were obtained by a humidity sensor. Over time, the level of the humidity outlet of membrane was closed to the inlet. When the levels of the humidity inlet and the outlet membrane were the same, it meant that the ILM had reached its saturation.

To compare the performance of the ILM for removing humidity, the mass of water absorbed by membrane can be obtained from the following equations:

(1) $$m_{water\;vaper\;absorbed}\left(g\right)=\overset{t_{saturated}}{\underset{t=0}{\int }}\left(Q_{inlet}-Q_{outlet}\right)\times M_{water}dt\;$$

(2) $$y=\frac{m_{water\;vaper\;absorbed}}{m_{IL\;used\;by\;membrane}}\times 100\%$$

where *Q_inlet_* is the molar mass of the water vapor inlet membrane, *Q_outlet_* is the molar mass of water vapor outlet from membrane (mol·min^−1^), *M_water_* is the molecular weight of water (*M_water_* = 18 g·mol^−1^), *t* is time (min), *m* is total mass of water absorbed by the ILM (g), and *y* is the percent mass of water absorbed per gram of IL.

## 3. Results and Discussion

In this study, the ionic liquid was used as a physical absorbent to separate water vapor from air. The ceramic tubular membrane acts as a hydrophobic support. Also, the effects of various parameters were test and the results were discussed.

### 3.1. Selection of Ionic Liquids

According to the absorbed water capacity, the ionic liquid was selected as an absorbent to fill the channels of a tubular ceramic membrane; [bmim]Br, [bmim][PF_6_], and [3htdp]Cl were chosen to test the abilities of humidity removal.

**Figure 3:**
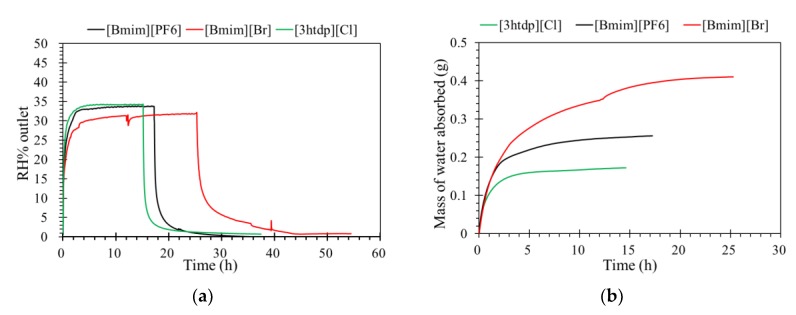
(**a**) The effect of ionic liquids filled into an ILM on its humidity separation performance; (**b**) the mass of water absorbed by an ILM with different ionic liquids. (relative humidity (RH)% inlet = 35%, temperature (T) = 32 °C, pressure = 1.0 atm, and flow rate = 0.5 L·min^−1^).

The levels of humidity outlet of the ILM as a function of time are shown in Figure 3a. When the [bmim][PF_6_] and [3htdp]Cl reached their absorption saturation, the level of humidity outlet by [bmim]Br was less than than 30%. This result confirmed that [bmim]Br has greater affinity with water than [bmim][PF_6_] and [3htdp]Cl [19]. Moreover, Figure 3b shows the mass of water absorbed by ILM. It more clearly shows that water is more soluble in [bmim]Br than in the others, which demonstrates that the water absorption by [bmim]Br is expected to be more effective. 

Because our membrane is a hydrophobic ceramic membrane, the water absorption is only dependent on its affinity with water of the groups compounding the ILs [8,12]. Normally, water can interact with both the cations and anions; therefore, the chemical structure of the ionic liquids would affect water absorption. The Br^−^ anion is a characteristic group of [bmim]Br that interacts with water molecules [12,20,21]. In the presence of water, the hydrogen bond involving the Br^−^ was enhanced. In addition, the anions preferentially interact with water, and since the anion promotes water molecule association through hydrogen bonds, the [bmim]Br was mainly responsible for water absorption. For the [bmim][PF_6_], according to the research of Cammarata et al. [22], water molecules dissolved in the anions are not self-aggregated water molecules interacting via hydrogen bonds with the anions in a symmetric complex (anion–H–O–H–anion). This means that water molecules do not interact with themselves, resulting in a lower sorption than [bmim]Br. There are longer alkyl chains in the cation of [3htdp]Cl, which could decrease the water solubility of the IL [15,16,17,18,21] and lead to a slower sorption rate in the future [19,23]. The impact of the anion types on the water absorption ability by ILs was found to be even stronger when compared to that of the cation types [23,24,25,26]. Thus, the absorption of water at 35% RH by the [bmim]Br, [bmim][PF_6_], and [3htdp]Cl was 0.072, 0.005, and 0.004 gram per gram of each of the ILs. Hence, based on our experimental data, the water absorption capacity of the ILs was as follows: [bmim]Br > [bmim][PF_6_] > [3htdp]Cl (Table 2). Therefore, [bmim]Br was selected and used to remove humidity in this study.

### 3.2. Inlet Flow Rates

**Figure 4:**
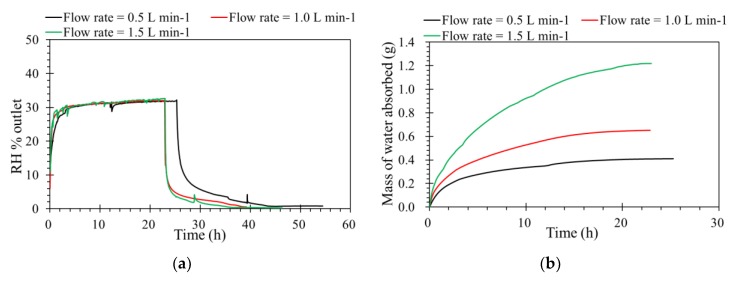
(**a**) The effect of flow rate on the humidity separation performance of the ILM. (**b**) The mass of water absorbed by the ILM with different flow rates. ([bmim]Br: RH% inlet = 35%, T = 32 °C, pressure = 1.0 atm).

Flow rate is one of the key operating factors affecting the performance of membrane processes. The tests were carried out from 0.5 to 1.5 L·min^−1^ of the feed flow rates, and the other operating parameters were constant. According to Figure 4a, levels of humidity outlet as a function of time. This shows that absorbing capacities are almost similar under different inlet flow rates, because the flow rate is affected the hydrodynamic gas channels. From the diffusion point of view, the driving force for water vapor removal is mainly determined by the differences in relative humidity and partial water vapor pressure across the membrane [27]. An ILM under a higher flow rate is faster to reach its absorption saturation. Even the contact time decreases with a flow rate increase from 0.5 to 1.5 L·min^−1^; the mass of water vapor sieving through the membrane increases with a higher feed flow rates, as shown in Figure 4b. For the same concentration, when the feed flow rate increased, the quantity of water feed increased, so the mass of water absorbed by the IL was higher during the same time before saturation. Furthermore, in order to verify the absorption process, the inlet RH and pressure were modified based on a constant inlet flow rate and concentration of RH.

### 3.3. Pressures and Relative Humidity

**Figure 5:**
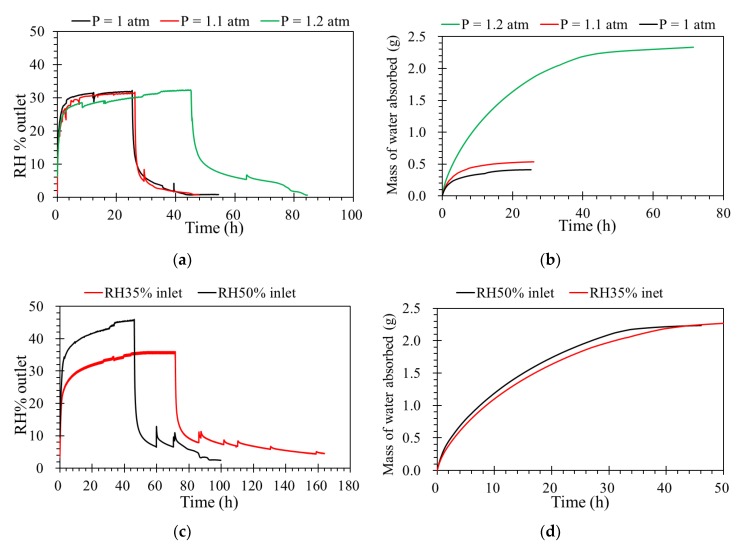
(**a**) The effect of absolute pressure on the humidity separation performance of the ILM. (**b**) The mass of water absorbed by the ILM under different absolute pressures ([bmim]Br: RH% inlet = 35%, T = 32 °C, flow rate = 0.5 L·min^−1^). (**c**) The effect of feed concentrations of relative humidity on the humidity separation performance of the ILM. (**d**) The mass of water vapor absorbed by the ILM ([bmim]Br, T = 32 °C, pressure = 1.0 atm, flow rate = 0.5 L·min^−1^)

To investigate the effect of differential pressures on humidity transport in the porous support, the ILM was registered at different pressures from 1.0 to 1.2 atm. One of advantage of vapor separation is that a much higher pressure can be applied across the membrane, which should translate into a higher driving force [28,29]. When the feed flow rate and concentration of humidity inlet are constant, from Figure 5a, at given feed flow rate, it can be clearly seen that the ILM under lower pressure is much faster to reach to its absorption saturation. Furthermore, before the absorption saturation, during the same time, higher pressure provides more water absorbed by ILM (Figure 5b). A higher driving force is provided by higher pressure, which results in a stronger diffusion during the porous ceramic membrane, and more water vapor reaches the ionic liquid and complete the absorption process.

The water vapor concentration inlet is another variable that can affect the membrane’s separation performance. In this case, when the inlet flow rate was constant, the higher feed concentration provided a higher driving force [30]. This effect is shown in Figure 5c. By increasing the water vapor concentration inlet, a higher amount of water vapor reaches the ILM provides a higher concentration gradient, which increases the driving force of mass transfer, resulting in the increase of mass transfer flux. According to Figure 5d, the mass of water absorbed with different feed concentrations of RH inlet are similar, while the total mass of water absorbed by the ILs were the same, even with different feed concentrations.

### 3.4. Temperature

**Figure 6:**
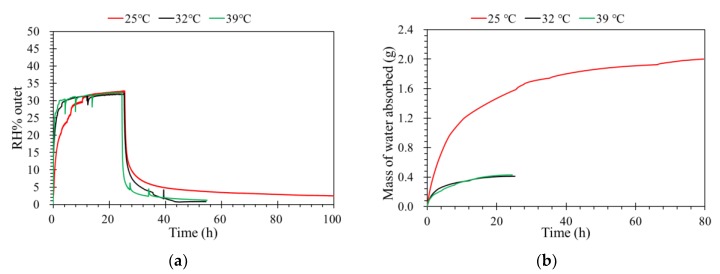
(**a**) The effect of temperature on the humidity separation performance of the ILM. (**b**) The mass of water absorbed by the ILM under different temperatures ([bmim]Br: RH% inlet = 35%, pressure = 1.0 atm, flow rate = 0.5 L·min^−1^).

Separation temperature is an important parameter for vapor separation. A higher temperature means a higher saturated vapor pressure for a certain feed, which means a higher driving force and flux. The diffusivity of gas molecules increases exponentially with temperature, and a higher flux is expected at higher separation temperature [26,31]. As shown in Figure 6a, when the operating temperature increases in the porous media, the thermal motion of water vapor molecules increases and the transfer resistance of supported ceramic membrane decreases [32], which results in a stronger diffusion. With the temperature increasing, the flux of water vapor in a gas channel increases, because the saturation pressure of the gas increases dramatically at higher temperature. Therefore, the vapor mass transfer from a gas channel to the ionic liquid channel increases due to the increased activity difference between the two channels.

Figure 6b displays the mass of water absorbed by the ILM over time, where the mass of water absorbed by the IL was higher when the ILM was registered at a lower temperature than at a higher temperature. The variation of the solubility versus temperature is a function of the solubility [33,34]. For low-solubility gases, the solubility will increase with increasing temperature, but for larger-solubility gases, the solubility will decrease with increasing temperature. For the ionic liquid used in this study, the maximum mass of 1.42 g water was absorbed by 10 g [bmim]Br, so the water vapor appears to be larger-solubility (1 g of solute dissolve into 10 g of solvent). Moreover, when the temperature increase, the vapor fraction was increased. This means when the temperature increases, the solute gas tends to be in the gas phase [35].

If we supposed that there was no phase transformation in the water vapor absorption by ionic liquid, the water always remains in the gas phase. When increasing temperature, the dissolved water will leave an ionic liquid (in the liquid phase). This can explain why we have a good regeneration of our membrane by increasing the temperature (see Section 3.6). It also means that the mass of water absorbed decreases with increasing temperature.

### 3.5. Comparison of ILM Performance with Different Operating Conditions

As shown in Table 3, the mass of water absorbed by the same ILM under different operating conditions was compared. The results show the feed concentration has the least influence on the mass of water absorbed. For the inlet flow rate, the mass of water absorbed by the ILM increased from 0.41 to 1.22 g when the inlet flow rate increased from 0.5 to 1.5 L·min^−1^. The mass increased by 0.81 g. The influence of pressure was a little bigger than the inlet flow rate. When the absolute pressure increased from 1.0 to 1.2 atm, the mass increased 0.97 g. The biggest influence on the mass of water absorbed by ILM was from temperature, which increased 1.57 g in total when the temperature decreased from 39 to 25 °C. Moreover, this room temperature proceeding limits the energy consumption.

The differences from flow rate, pressure, and feed concentration were only affected by the kinetic process, and the total mass of absorption by the ILM was only dependent on the quantity and nature of the IL. Compared to the other operating parameters, the temperature was mainly affected by the solubility of water in the IL [33,34,35]. This indicates that when the temperature increases, the total mass of absorption by the ILM decreases, and ILM cannot reach the same saturation capacity even under optimal conditions.

### 3.6. Position of Gas Channels

**Scheme 1:**
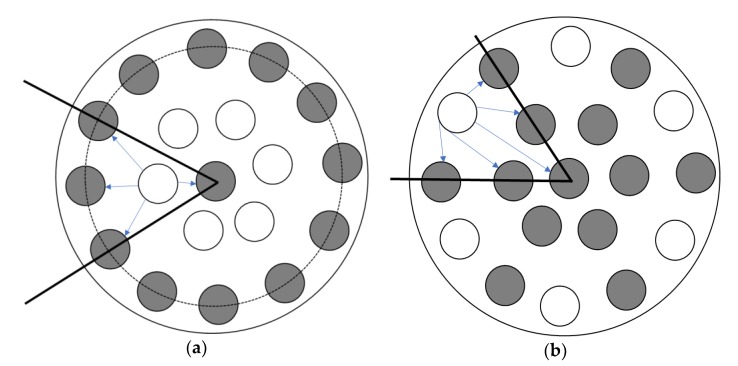
The position of the gas channels (channels with IL are gray, channels without IL are white).

**Figure 7:**
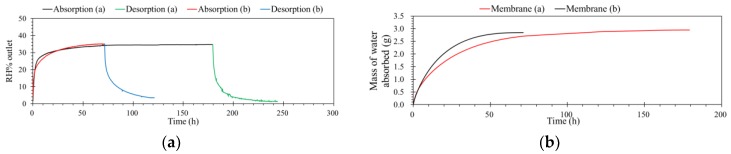
(**a**) The effect of gas channel position on the humidity separation performance of the ILM. (**b**) The mass of water absorbed by the ILM with the different position of gas channels ([bmim]Br: RH% inlet = 35%, pressure = 1.0 atm, temperature = 32 °C, flow rate = 0.5 L·min^−1^).

The position of the gas channels can also affect the separation performance of ILM. As shown in Scheme 1, the cross-sectional schemes of ILMs show that the number of channels for the IL and feed stream are the same. In this case, when the membranes contained the same mass of IL ([bmim]Br), for the absorption, the surface areas were also same. More details of membranes are exhibited in Table 4. The membranes were registered at same conditions, including level of RH inlet, pressure, temperature, and flow rate. According to Figure 7a, Membrane (b) was much faster to reach its absorption saturation than Membrane (a). When the level of humidity outlet was equaled to the inlet, for Membrane (a) it took 180 h, while for membrane (b), it only needed 71 h. This result demonstrates that the position of the gas channels is influenced the separation performance of the ILM. Furthermore, based on the mass of water absorbed by the ILMs, Membrane (b) can obtain more water within a shorter time than Membrane (a), which suggests that the Membrane (b) can remove humidity from a feed stream faster and more effectively. However, when these two membranes reached their absorption saturations, the total mass of water absorbed was almost equal (2.9 ± 0.5 g) (Table 4). Results showed that the position of the gas channels only affects the absorption rate at the beginning—there is no effect on the final target.

As shown in Scheme 1, each gas channel was researched in the early stage of absorption. On average, for Membrane (a), of the outermost IL channels, only half can be regarded as active, and the other half is not working at the beginning of absorption. Oppositely, for Membrane (b), all the IL channels can be regarded as active. Moreover, the directions of humidity diffusion for Membrane (b) are much greater than Membrane (a). These give a good explanation for the position of gas channels only influencing the early stage of absorption, but not the final target.

### 3.7. Regeneration

**Figure 8:**
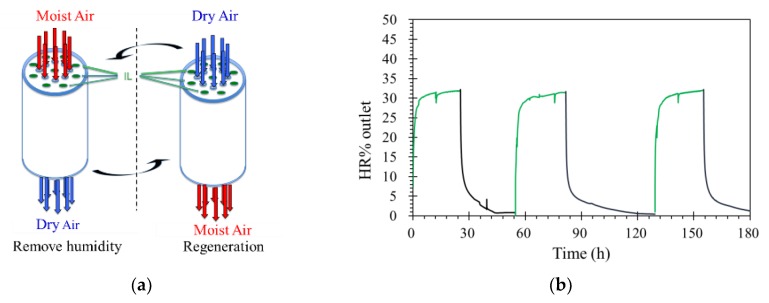
(**a**) The schematic diagram of removing and regeneration processes. (**b**) Three-time recycling of the ILM ([bmim]Br: RH% inlet = 35%, remove humidity (green)/RH% inlet = 0 regeneration (black), T = 32 °C, pressure = 1.0 atm, flow rate = 0.5 L·min^−1^).

Processes for the regeneration of rich water-loading ILs were operated in the same unit with same conditions, but only the feed stream was changed, from contained humidity to dry zero air. The flow rate was constant at 0.5 L·min^−1^, with 1.0 atm and 32 °C. When carried on the desorption process, the concentration of the feed just changed from 35% to 0% RH. Figure 8a shows the details of this operation. As shown in Figure 8b, results indicate that the water contents of ILs decreased with time under the dry air flow. After three times absorption and desorption, the capacity of ILM absorption was not decreased. Furthermore, there were no drops of IL observed, even under high temperature for long-time operation. This means that this process is simple and efficient.

## 4. Conclusions

IL-based membrane processes have been widely applied to remove volatile organic compounds (VOCs) and CO_2_, while removal of humidity by this process is still in progress. A new ILM that combinates the separation process of a ceramic membrane and the absorption process of IL was developed, with the objective of removing humidity from air. The air dehumidification efficiency of ILM was compared using three ionic liquids, including [bmim]Br, [bmim][PF_6_], and [3htdp]Cl. According to the quantity of water absorbed by ILM, the hydrophilic ionic liquid [bmim]Br was selected. 

Additionally, the influences of operating conditions, such as flow rate, temperature, pressure, and feed concentration, on the separation performance of ILM were investigated. Results indicate that when the flow rate increases, both mass transfer and hydrodynamics are promoted. However, the contact time of humidity and ILM decreased, which results in a weakened mass transfer. The feed concentration only affected the absorption rate, but not the total mass. When the temperature of the operating system increases, the efficiency of the membrane on humidity removal decreased. However, the processes were operated at room temperature, due to the limitation of energy consumption. The regeneration process is simplified and available, and just needs to change the feed stream from contained humidity to dry air. After three-time recycling, the absorbing capacity of ILM was not decreased. Moreover, there were no loss of IL observed under high pressure and temperature. IL-based membrane processes will be the dominating green processes for humidity separation.

## 5. Patents

Anguille S.; Moulin P. Device for extraction of pollutants by multichannel tubular membrane, EP 14306002.8, 2016, EP2015/064462.

## Figures and Tables

**Table 1:** Specifications of the ILM.

Effective length (cm)	25
Channels size (mm)	2
Total number of channels	52
Number of channels with IL	32
Surface area (m^2^)	4.0 × 10^−2^
Molecular weight cut-off (Da)	1000

**Table 2:** Chemical structures of the ionic liquids (ILs) used.

Content	[bmim]Br	[bmim][PF_6_]	[3htdp]Cl
Name	1-butyl-3-methylimidazolium Bromide	1-butyl-3-methylimidazolium Hexafluorophosphate	Trihexyl(tetradecyl)phosphonium Chloride
Chemical structure	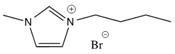	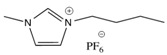	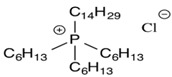
Molar mass (g·mol^−1^)	219.13	284.19	519.32
Water affinity	hydrophilic	hydrophobic	hydrophobic
Mass of IL used (g)	49.0	53.4	34.1
Mass of water absorbed by per gram of IL (g)	0.14	0.033	0.055

**Table 3:** Summary of the mass of water absorbed by the same ILM under different operating conditions.

Operating Conditions		Mass of Water Absorbed (g)	*y* (%)
Flow rate (L·min^−1^)	0.5	0.42	0.86
	1.0	0.65	1.33
	1.5	1.22	2.59
Pressure (atm)	1.0	0.42	0.86
	1.1	0.53	1.08
	1.2	2.33	4.76
Feed concentration (%)	35	2.34	4.78
	50	2.33	4.76
Temperature (°C)	25	2.00	4.08
	32	0.41	0.84
	39	0.43	0.88

**Table 4:** Summary on the position of the gas channels.

Content	Membrane (a)	Membrane (b)
Total channels	19	
Channels with IL	13	
Channels without IL	6	
Mass of IL (g)	38.7	38.4
Total mass of water absorbed by ILM (g)	2.95	2.85
